# Supplementary figures and images for: The LHX2-OTX2 transcriptional regulatory module controls retinal pigmented epithelium differentiation and underlies genetic risk for age-related macular degeneration
Source: PLoS Biol. 2023 Jan 17;21(1):e3001924. doi: 10.1371/journal.pbio.3001924 (PMC9844853; doi:10.1371/journal.pbio.3001924)

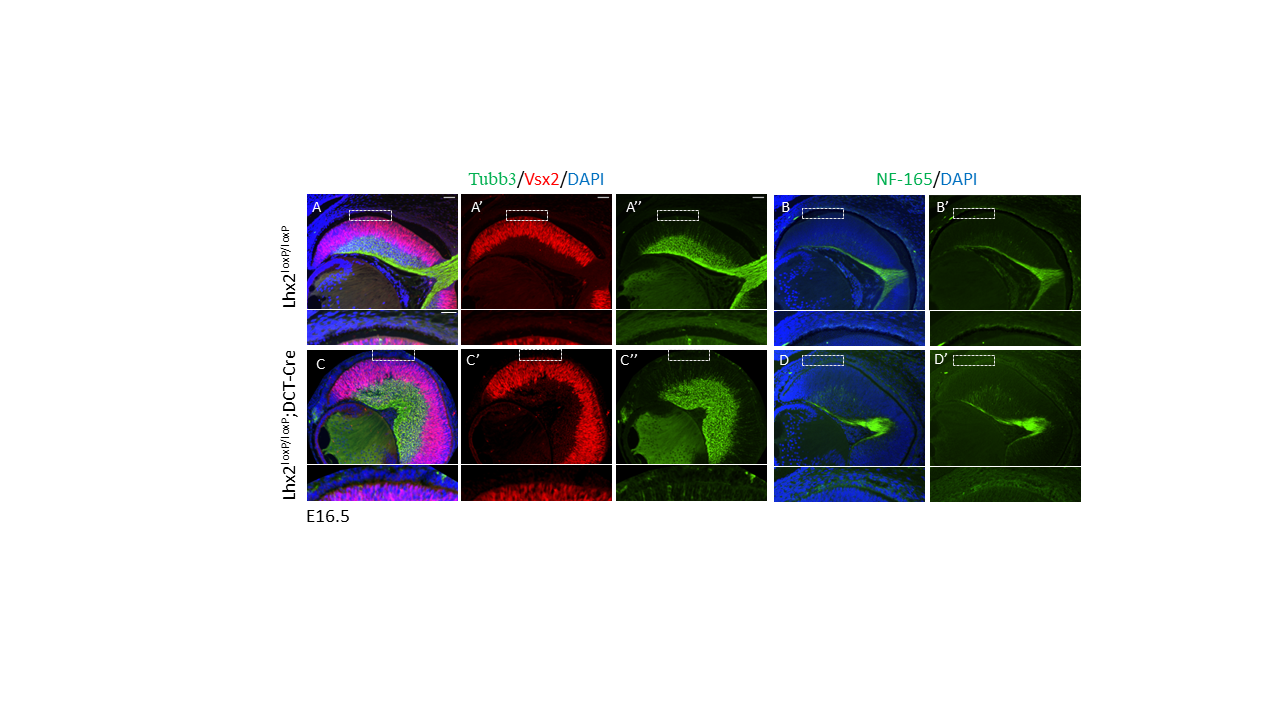

Supplement: S1 Fig — In E16.5 control (A, B; A’, A”, and B’ are the respective separate channels) and Lhx2loxP/loxP;DCT-Cre (C, D; C’, C”, D’ are the respective separate channels) developing RPE the expression of neuronal markers Tubb3, Vsx2 (A, C), and NF-165 (B, D) are not detected by indirect immunofluorescent analyses. DAPI was used for counterstaining of the nuclei. Scale bar is 50 μm, lower insets are 10 μm. (TIF) [file pbio.3001924.s001.TIF]

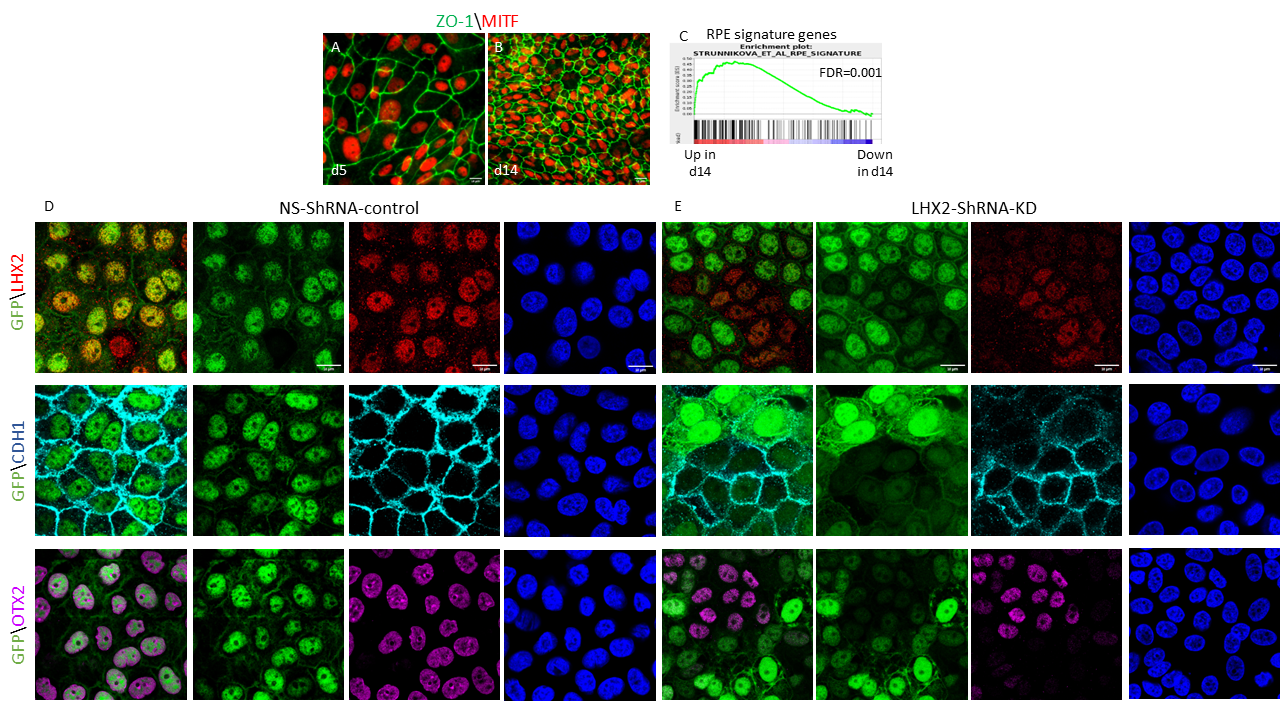

Supplement: S2 Fig — Expression of tight junction protein ZO-1 (green) and the transcription regulator MITF (red) in (A) d5 and (B) d14 culture of hES-RPE. Scale bar is 10μm. (C) Gene set enrichment analysis comparing gene expression profiles in differentiated (d14) and de-differentiated (d5) hES-RPE cells shows a significant enrichment for the “RPE gene signature” among the genes up-regulated in d14. (D) The lentiviral transduction of control NS-shRNA or (E) LHX2-shRNA KD to hES-RPE (d14). GFP (green) marks the transduced cells. The indirect immunofluorescent analyses were conducted with antibodies against LHX2 (red, top row), CDH1 (blue, middle row), and OTX2 (purple, lower row). Composite shown on right; adjacent are the separate channels. DAPI (blue) labels the nuclei. Scale bar is 10 μm. (TIF) [file pbio.3001924.s002.TIF]

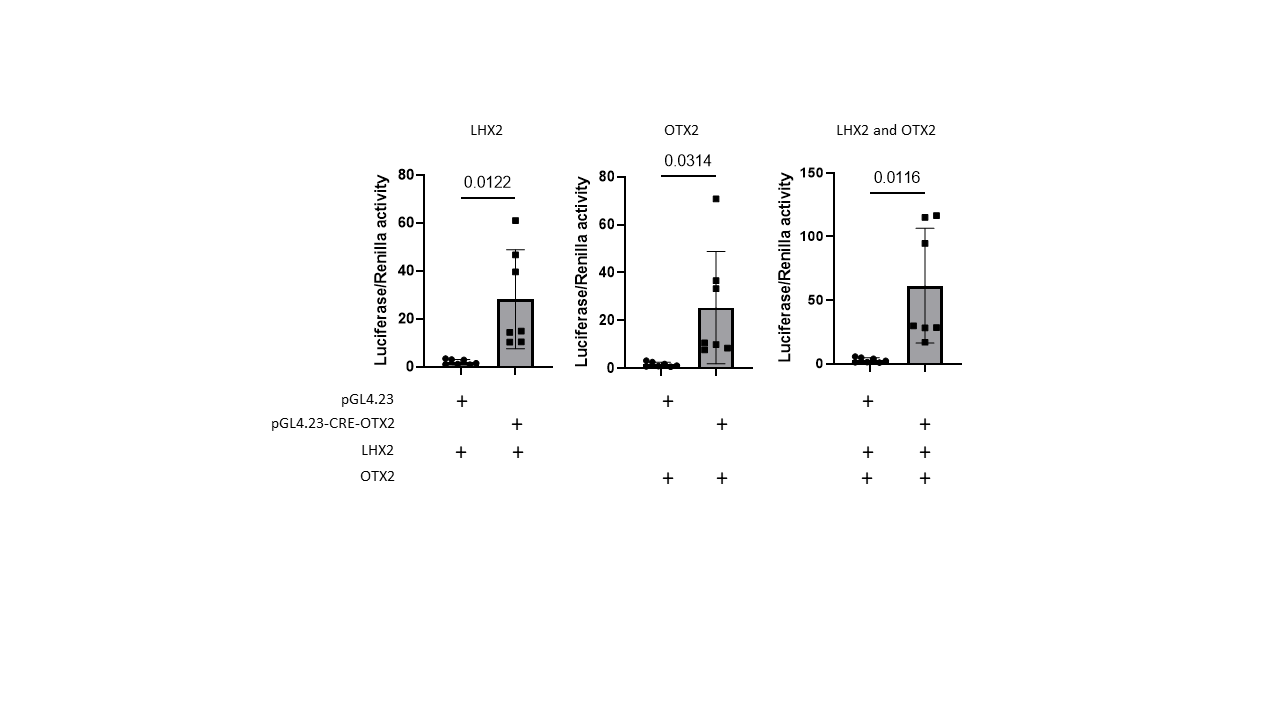

Supplement: S3 Fig — Luciferase reporter activity induced by LHX2 or OTX2 or both on the PGL4.23-OTX2-CRE as compared to the TFs activity on empty PGL4.23. The p-values calculated by t test, two-tailed, paired (N = 7, the data and analyses are detailed in S10 Table). (TIF) [file pbio.3001924.s003.TIF]

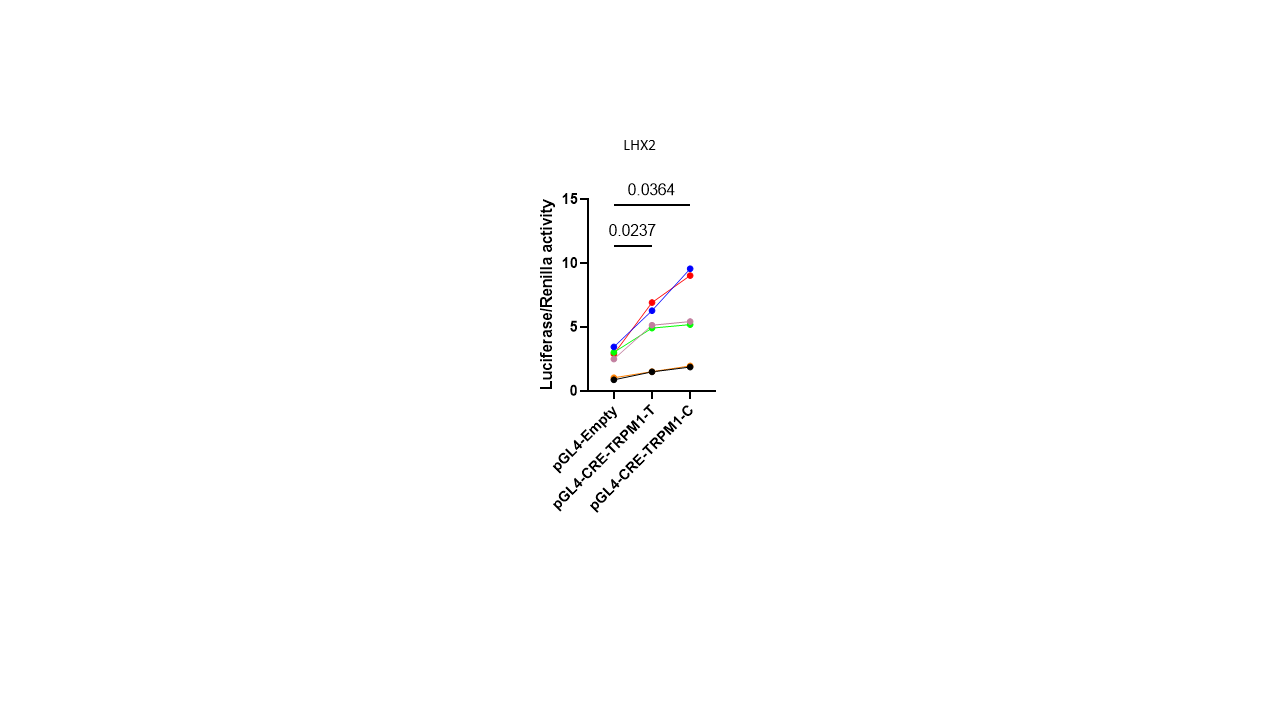

Supplement: S4 Fig — Luciferase reporter activity induced by LHX2 overexpression on 309 bp that contain regulatory element bound by LHX2 in rs3809579. Both CRE-TRPM1-T and CRE-TRPM1-C alleles are significantly activated by LHX2 as compared to its activation of empty PGL4.23. The p-values calculated by one-way ANOVA, matched samples, corrected with Dunnett’s for multiple comparisons (N = 6). In all 6 independent experiments, transcriptional activity of the CRE-TRPM1-C is higher than CRE-TRPM1-T (overall p-value of this consistent trend is 0.56 = 0.016; binomial test). The data underlying this figure are detailed in S10 Table. (TIF) [file pbio.3001924.s004.TIF]
