# Supplementary material for: The LHX2-OTX2 transcriptional regulatory module controls retinal pigmented epithelium differentiation and underlies genetic risk for age-related macular degeneration
Source: PLoS Biol. 2023 Jan 17;21(1):e3001924. doi: 10.1371/journal.pbio.3001924 (PMC9844853; doi:10.1371/journal.pbio.3001924)
Supplement: S1 Raw image — The 293T cells overexpressing OTX2, LDB1, and LHX2 in the indicated combinations (Input) were subjected to immunoprecipitation with Flag antibodies followed by western blot analysis and immunolabeling with OTX2 antibody. (PDF) [file pbio.3001924.s005.pdf]

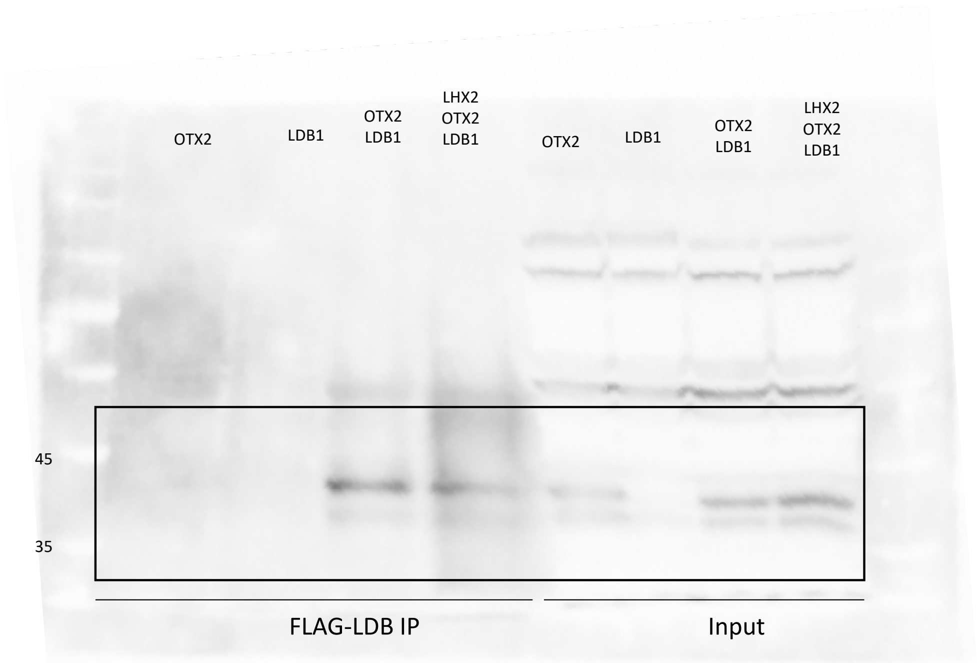

Immunoblot for OTX2 of immunoprecipitants with FLAG antibody (FLAG-LDB1), original blot presented in Fig 5B
