# Supplementary material for: The LHX2-OTX2 transcriptional regulatory module controls retinal pigmented epithelium differentiation and underlies genetic risk for age-related macular degeneration
Source: PLoS Biol. 2023 Jan 17;21(1):e3001924. doi: 10.1371/journal.pbio.3001924 (PMC9844853; doi:10.1371/journal.pbio.3001924)
Supplement: S8 Table — (DOCX) [file pbio.3001924.s013.docx]

Table S8

| **Antigen** | **Source** | **Manufacturer** | **# Catalog** | **Dilution** |
| --- | --- | --- | --- | --- |
| Tubulin Beta 3  (Tubb3/Tuj1) | mouse | Chemicon | mab1637 | 1:500 |
| E-cadherin  (CDH1) | mouse | BD transduction laboratories | 610182 | 1:250 |
| GFP | goat | Abcam | ab6673 | 1:200 |
| Lhx2 | Goat | Santa Cruz | Sc-19344 | 1:50 |
| Mitf | mouse | [1] | (Clone C5, kindly provided by Dr. David. E. Fisher and Carmit Levy), | 1:50 |
| Nf165  (NEFM) | Mouse | Hybridoma Bank | AB 2314897 | 1:500 |
| Otx2 | Rabbit | Abcam | ab21990 | 1:250 |
| Pax6 | Rabbit | Biolegend | 901301 | 1:100 |
| P-cad  (CDH3) | Rat | Invitrogen | 132000z | 1:800 |
| Sox9 | Rabbit | Chemicon | ab5535 | 1:200 |
| Vsx2 | Sheep | Exalpha | X1180P | 1:1000 |
| ZO-1  (TJP1) | mouse | Invitrogen | 33-9100 | 1:100 |
| **Secondary antibodies** |  |  |  |  |
| anti-Rat -488 | Goat | Invitrogen | A11006 | 1:1000 |
| anti- Rabbit -594 | Donkey | Invitrogen | A-21207 | 1:1000 |
| anti- Mouse -488 | Donkey | Invitrogen | A-21202 | 1:1000 |
| anti- Goat -594 | Donkey | Invitrogen | A-11058 | 1:1000 |

Reference

1. Malcov-Brog H, Alpert A, Golan T, Parikh S, Nordlinger A, Netti F, et al. UV-Protection Timer Controls Linkage between Stress and Pigmentation Skin Protection Systems. Mol Cell. 2018;72(3):444-56 e7. Epub 20181025. doi: 10.1016/j.molcel.2018.09.022. PubMed PMID: 30401431; PubMed Central PMCID: PMCPMC6224604.
