# Supplementary material for: The LHX2-OTX2 transcriptional regulatory module controls retinal pigmented epithelium differentiation and underlies genetic risk for age-related macular degeneration
Source: PLoS Biol. 2023 Jan 17;21(1):e3001924. doi: 10.1371/journal.pbio.3001924 (PMC9844853; doi:10.1371/journal.pbio.3001924)
Supplement: Attachment — Submitted filename: reviewers comments_response_14092022.docx [file pbio.3001924.s016.docx]

**Reviewer #1:** This is a very interesting paper dealing with transcriptional regulatory networks important for the retinal pigment epithelium of the eye.

The authors combined mouse in vivo data with human RPE datasets for RPE regulatory regions and defined an interplany between LHX2 and OTX2.

Matching this LHX2-OTX2 cistrome (ChIP-Seq) with ATAC-seq data for open chromatin regions and ultimately with GWAS SNP data for age-related macular degeneration, the authors make a clear point that the regulatory region of the AMD risk gene TRPM1 seems to be under risk SNP-dependent regulatory control.

The group invested a lot of work and did a great job in applying sophisticated methods important for regulatory network analysis.

I have only a few minor points of criticism that would balance the paper´s scope.

**Response:** We thank the reviewer for the appreciative comments and positive feedback on the analysis and the impact of our study, and for the helpful comments for revision. Accordingly, we have corrected the manuscript and introduced the following changes, as explained in detail after each comment.

**1 -**The RPE is in a tight connection with the underlying retina. Both LHX2 and other factors including Crx have been well studied in transcriptomic analyses e.g. using ChIP-Seq. Relevant papers, e.g. the first ChIP-Seq study on retinal cells (Corbo et al, 2010) should be discussed.

**Response:** Page 17 – The following has been added: “This approach has been instrumental for determining cell type specific cis regulatory regions of Otx2, Crx and Lhx2 in retinal progenitor and retinal lineages [38, 62, 63]. Here we mapped the LHX2 and OTX2 bound sites in hES-RPE. ”

**2** -The in vivo mouse model for LHX2 (Figure 1) needs a better explanation why this promoter was used for cell specific KO and also a bit more background on LHX2 in general.

**Response:** Page 6, first paragraph – We have added the following details and clarifications on the mouse lines:

“To directly examine the roles of *Lhx2* *in vivo* in specified RPE progenitors, we conditionally deleted *Lhx2* from the PE progenitors using the *Lhx2^loxP^* line [30] and *Dct-Cre* (previously termed *Tyrp2-Cre* [31]). *Dct-Cre* is active in the specified PE progenitors of the optic cup starting with onset of RPE differentiation around embryonic day 10 (E10) [31-33]. The analyses were conducted on *Lhx2^loxP/loxP^;Dct-Cre* (termed *Lhx2*-PE-cKO) mice and control litter mates that do not carry the *Dct-Cre*.”

**3**-I could not find that the newly generated large scale sequencing datasets were deposited in public databases.

**Response:** The omics data generated by our study is now available at GEO. We have added this to the Methods section.

To review GEO accession GSE178166 (private view until publication), go to:

https://www.ncbi.nlm.nih.gov/geo/query/acc.cgi?acc=GSE178166

and enter token kzoreogwdpitjwp

**Reviewer #2:** The manuscript studied the gene regulatory networks centering the transcription factor LHX2 in retinal pigment epithelium (RPE) cells, and used the information to uncover causal non-coding regulatory risk SNPs for AMD. The authors first showed that LHX2 is required for the maintenance of RPE genes in mice RPE in vivo and in differentiated human RPE. They then found that LHX2 and OTX2 co-occupied on cis-regulatory elements and form a complex with chromatin remodeling factors to collectively regulate RPE gene expression. Lastly, they identified an AMD risk SNP that affects TRPM1 regulation by the LHX2-OTX2 complex by intersecting published ATAC-seq, GWAS and genomic datasets.

The manuscript is of interest to readers in the field of genetics and genomics who study gene regulatory networks and their association with diseases. While the primary claims are well supported by the data, additional investigation and explanation are necessary to make the study more convincing.

**Response:** We are grateful for the positive evaluation and the constructive comments. We address the comments and introduced the following changes, as explained in our point-by-point responses below.

**Major concerns:**
1. The manuscript mostly focused on the role of Lhx2 in maintaining RPE gene expression (in differentiated human RPE). But the first part of the results focused on Lhx2's function in specifying RPE. This seems to be a little disconnected. Have the authors tried to delete Lhx2 in mature RPE in mouse in vivo and assess its function in maintaining RPE genes?

**Response:**

We did invest a great deal of time and effort in attempting to mutate *Lhx2* in the adult mouse RPE using the RPE-Tyrosinase-CreErT2 line. To our disappointment, we failed to obtain Cre activity with this line in the adult RPE, possibly due to an epigenetic mechanism leading to silencing of the transgene. Obtaining a new founder/different line would require extensive investment of time and budget. Please note that a role for OTX2 in adult RPE has been previously reported and we did observe significant reduction in the reported Otx2 targets in {Housset, 2013 #264} among the down regulated genes following LHX2 KD in hES-RPE (Fig. 3A and new tab in revised Table S2). This provides support for conservation of the proposed regulatory hierarchy in mice as well. As also noted by this reviewer, the current study focuses on the LHX2–OTX2 regulatory module in human RPE and thus studies on Lhx2’s role in the adult mouse RPE, although planned for future work, are beyond the scope of the current report.

2. The authors claimed that LHX2 does not directly interact with OTX2 but forms a complex with OTX2 via LDB1. However, it is also possible that OTX2 and LHX2 form complexes with LDB1 independently. The OTX2/LDB1 and LHX2/LDB1 complexes bind to neighbouring regions in the genome without real physical interactions. The authors need to provide additional information if they claim that OTX2 and LHX2 are in the same complex.

**Response:** We agree that further experiments are required to support the physical association between LHX2 and OTX2, and how this is influenced by LDB1. We therefore replaced, throughout the manuscript the term “complex”, which refers to a physical association, to “regulatory module”, which refers to a functional interaction between LHX2 and OTX2 in transcriptional regulation. We modified the title and running title accordingly: “The LHX2–OTX2 **regulatory module** controls RPE differentiation and underlies a causal regulatory risk SNP in age-related macular degeneration”.

The running title: **LHX2-OTX2 regulatory module in RPE differentiation and AMD**

**Our findings, however, do provide evidence that LHX2 and OTX2 function together on a shared** cistrome (cis regulatory elements, CREs). This is based on: 1. the identification of co-bound genomic regions (close to 70% of the LHX2-bound sites are co-occupied by OTX2 based on ChIP-seq, Fig. 3D). Importantly, the genes associated with the bound sites (based on proximity) are significantly reduced in the LHX2-KD hES-RPE (Fig. 2K), and known targets of Otx2 in the mouse RPE are reduced in the LHX2-KD hES-RPE (Fig. 3A); 2. the finding that both interact with LDB1 based on IP-MS analyses (Fig. 5A), further supporting a shared mechanism in gene regulation. This is because LDB1 is a well-documented partner of LHX2 (and other LIM domain proteins) and its interaction with OTX2 (validated by co-IP, Fig. 5B) does infer a role in at least some of the co-bound CREs.

Finally, in the revised manuscript we have added experimental support for the shared activity of LHX2 and OTX2. A luciferase reporter assay was conducted on a co-bound regulatory region that was previously identified to drive reporter expression in vivo in an Otx2 pattern of expression, including in the RPE (hs1150 [41] [42]. We assayed the functional interaction between LHX2 and OTX2 on this enhancer (termed CRE–OTX2; new Fig. 4C and D). CRE–OTX2 was significantly induced (new Fig. S3) by either *LHX2* (~13-fold *p* = 0.012, N = 7) or *OTX2* (~14-fold, *p* = 0.03, N = 7) overexpression. The relative reporter activity was further enhanced by the overexpression of both LHX2 and OTX2 (~21-fold, *p* = 0.01, N = 7, Fig. 4D, shows the over expression of both). The latter finding further supports the shared, cooperative (rather than competing) transcriptional activity of LHX2 and OTX2 on CRE–OTX2.

3. Combining LHX2/OTX2 ChIP-seq data with available GWAS and ATAC-seq datasets to identify non-coding risk variants is very interesting and could have wider impact in the field. However, the author didn't verify their findings experimentally. For example, would LHX2 activate a reporter construct containing the TRPM1 variant in differentiated human RPE?

**Response:** We thank the reviewer for this important comment. We have added a luciferase reporter assay in RPE cell line D407 to examine regulation of the identified CRE for TRPM1 by LHX2. We show that indeed, this regulatory element is activated by LHX2 (Fig. S4). Importantly, in accordance with our model, we show that this activation is stronger for the construct with the C allele of this risk variant than with its T allele (new Fig. 6G, *p* = 0.02).

4. RNA-seq results were not verified by qPCR, RNA in situ or protein staining.

**Response:** Reduced protein staining following Lhx2 conditional knockout in the mouse RPE is presented in Fig. 1 and the reduction of CDH1, LHX2 and OTX2 following LHX2 KD in the hES-RPE is presented Fig. 2A–F. The antibodies that we obtained for TRPM1 did not work for us. In any case, (1) the expression of TRPM1 was reduced ~18-fold upon LHX2 KD based on the RNA-seq data (*p* < 7.5 x 10^-11^ by DESeq2) (Fig. 6F) and (2) TRPM1 is among the genes shown to be downregulated following Otx2 inactivation in the mouse RPE (based on ([14], See Table S2 tab).

**Reviewer #3**: The authors describe a set of experiments aimed at studying the role of transcription factors LHX2 and OTX2 in retinal pigment epithelium development and AMD disease development. They use transcriptome profiling in a conditional Lhx2 KO mouse model and Knockdown protocol in well-defined RPE model of differentiated human embryonic stem cells (hES-RPE). Chip-seq experiments in the hES-RPE cells are described to identify LHX2 and OTX2 genomic binding sites. From the observed regulation of OTX2 expression by LHX2 and the spatial association of the LHX2 and OTX2 putative genomic binding sites, the authors suggest that these TFs function jointly in the RPE. Since they are unable to demonstrate direct interaction between the two TFs they suggest that they both bind a third factor, LDB1. They also identify a single nucleotide polymorphic (SNP) that was described as an expression-quantitative trait locus (eQTL) that changes a putative LHX2 binding motif with a LHX2/OTX2
ChIP-seq defined binding site (peak). Based on analyses of existing RPE ATAC-seq data they conclude that the base change in the SNP change the LHX2 affinity to the locus which results in reduced expression of the target gene, TRPM1, and increased AMD risk.

This manuscript describes a significant amount of very interesting work. However, it includes several deficits that should be corrected before publication. Specifically, some of the experiments are poorly described, the figure legends are poorly written and some of the conclusions are not well supported by the data provided.
Below are some comments that may help improve the manuscript.

**Response:** We are grateful to the reviewer for the positive evaluation and thorough and constructive review of the manuscript. We have corrected the manuscript as explained in detail below.

1. Page 13, last sentence of the first paragraph. Where is the data supporting higher affinity of LHX2 for the putative binding site with the C allele? Please describe the experiment that show that the C allele is associated with higher expression level of TRPM1 transcripts. Is this data from human RPE cells?

**Response**: Several lines of evidence support higher affinity of LHX2 to the binding site with the C allele: first, the C allele fits the documented binding motif of LHX2 (revised Fig. 6C). Second, eQTL analysis for the two alleles showed that the C allele is associated with higher expression of the target gene TRPM1 (revised Fig. 6A, eQTL data from Orozco et al., 2020). Third, the ATAC-seq data imply that the chromatin in this genomic region is more open (thus likely, more active) with the C allele (Fig. 6E).

To further address the reviewer’s concern about the impact of the C/T variants on LHX2 transcriptional activity, we conducted a luciferase reporter assay to examine LHX2 activation of the regulatory region spanning rs3809579 with either C or T alleles. This analysis showed that both alleles are activated by LHX2—significantly more than the baseline activity of the control empty vector (new Fig. S4). Importantly, the construct with the C allele showed stronger luciferase induction than the one with the T allele (Fig. 6G, *p* = 0.02). This result provides strong experimental support for the notion that LHX2 transcriptionally activates the C allele more strongly than the T allele.

2. The association of the increased AMD risk eQTL tag SNP rs3809579 with the ChIP-seq and ATAC-seq signals is interesting. However, very little data is provided to support a causal roll for this variant in the regulation of TRPM1 expression. The data includes 2 ATAC-seq samples with very few reads. To substantiate this observation the authors should introduce the C allele into the hES-RPE cells model, that will allow for direct assessment of the contribution of the C allele to LHX2 binding affinity, TRPM1 expression, and chromosome accessibility.

**Response:** We do not currently have access to additional RPE cells which are heterozygous for the C/T alleles of this risk SNP. Obtaining such lines from a repository or by genome editing is a task for future analyses in the laboratory that will require time and effort, beyond the scope of the current study.

In the revised manuscript, to establish a causal effect for this SNP, we experimentally evaluated the impact of its C/T alleles on LHX2 transcriptional activity by luciferase reporter assay (see our response to the previous point).

3. The analysis of the ATAC-seq data is lacking. For example, have the authors removed duplicated fragments form the analysis?

**Response:** We thank the referee for this comment. We repeated the allele-specific analysis of the ATAC-seq data after removal of duplicate reads, and this is now detailed in Methods; the results shown in Fig. 6E have been adjusted. In this new analysis as well, we observed 3 times more reads with the C allele than the T allele for both heterozygous donors.

Figure3:
1. Explain the schematic in figure 3A. What data was used to create this figure? What is the meaning of the different symbols? The description of this experiment in the result section is not clear.

**Response:** We decided to remove Fig. 3A from the revised version, because downregulation of Otx2 target genes in the LHX2-KD cells is strongly demonstrated in Fig. 3B (3A in the revised manuscript). The list of Otx2 targets is based on {Housset, 2013 #264}, and their level of suppression in the LHX2-KD hES-RPE is provided in a tab in Table S2.

2. Explain the data presented in figure 3C. This should probably only be mentioned in the results section. What were the results for the LHX2 ChIP-seq peaks?

**Response:** Fig. 3C (3B in the revised manuscript) shows the most highly enriched motif as detected by de-novo motif analysis on the set of our OTX2 ChIP-seq peaks. It corresponds to the known consensus binding motif of this transcription factor. We now explain this better in the figure’s legend. Results of de-novo motif analysis on the LHX2 peaks are presented in Fig. 2J.

3. Explain why the OTX2 motifs presented in figure 2J, figure 3C, and figure 3F are different. Similarly, the LHX2 motifs in figure 2J, figure 3F and figure 5C are different.

**Response:** There are some differences in the output of different programs with respect to motif size and orientation. We now present the motifs in a consistent manner. We have removed Fig. 3F and its information is now presented in the Results. The consensus LHX2 PWM is taken from Jaspar DB [104] (accession: MA0700.1)

4. Description of panel F figure 3 is missing.

**Response:** We have removed this panel from the figure. The results are presented on page 10, first paragraph.

Figure 4:
1. Please define "tars" in figure 4a.

**Response:** This stood for “targets”. We have changed this in Fig. 4A.

2. A basic explanation of the network examination using ingenuity pathway analysis is missing from the results section and from the figure legend.

**Response:** We decided to remove the IPA as it is redundant with the results presented in Fig. 3B (now Fig. 3A).

3. The western blot in figure 3 D is of low quality. The authors should provide a better-quality blot to support direct interaction between LBD1 and OTX1. The high background in the OTX2 and LBD1 IP lanes should be addressed. What is the rational of adding LHX2 to the experiment?

**Response:** We replaced the figure with a better quality blot. OTX2 is specifically immunoprecipitated by Flag-LDB1 with or without LHX2. Initially, we considered the possibility that the binding of LDB1 to OTX2 is dependent on the formation of a complex with LHX2, but the results support a direct association of OTX2 with LDB1. This association is neither dependent on, nor prevented by LHX2.

4. Figure 5:
1. Is the data presented in panel A from this study? If not, it should be referred to in the results or discussion sections with reference, not reproduced in a major figure.

**Response:** The data in panel A of Fig. 6 (in the revised manuscript) present our analysis of the eQTL for rs3809579. The figure was obtained using a web resource provided by Orozco et al. (2020). This result is important for establishing the link between the genotype of this SNP and the expression level of TRPM1. Thus, we strongly feel that it is important that this result be kept in the main figure, as it provides the starting point for our subsequent analyses in the section: delineating the mechanism of action of this variant and establishing it as the causal variant in this AMD locus (Fig. 6E–G).

2. In B, please explain the various boxes. What exactly are ATAC-seq D1 and ATAC-seq D2?

**Response:** We have added explanations to the legend of revised Fig. 6:

**“**An overview of the TRPM1 genomic region including the upstream regulatory regions, the conservation in vertebrates and zoom to the rs3809579 variant (underlie in red), which is located within LHX2 and OTX2 ChIP-seq peaks in the promoter of TRPM1, as well as ATAC-seq tracks from RPE of two human donors (D1 and D2)”

3. The schematics in C and D seem excessive.

**Response:** The schematics are required to present the two variants in the LHX2 motif (C) and to explain the allelic imbalance analysis (D) We believe that they are important for readability of this section and make it easier for readers to follow the analyses that we conducted and our findings. Therefore, we have opted to keep them in the main manuscript.

**Reviewer #4**: The manuscript of Cohen et al reports the genomic occupancy and functional role of the Lhx2 and Otx2 transcription factors in retinal pigmented epithelium. This data led to the hypothesis that a previously identified genetic variant associated with age-related macular degeneration is causal due to its decreased Lhx2 occupancy. Overall this work presents an exciting story with advances made in basic biology but additionally with a link from that data to a prevalent human disease. A strength is found in the diverse set of techniques used to generate the data to build the case for the hypothesis. However, there are further experiments that are required to fully support the conclusions that the manuscript leads to. The most important issue is to provide experimental evidence that the identified polymorphism does in fact affect Lhx2 binding and transcriptional activity.

**Response:** We are grateful to the reviewer for the positive and thorough evaluation of the manuscript. We have corrected the manuscript as explained in detail below.

**Major**
1. The link between the genomic occupancy of Lhx2 and the previously published eQTL data has several correlation links, but would be greatly strengthened by more empirical support to strengthen the disease-relevant conclusion. While the "C" in the first nucleotide position of the Lhx2 motif is certainly more represented in the Lhx2 consensus motif (note that the authors need to provide specifics on where this motif is from - a previous study, a particular cell type from ChIP, Selex, etc), it is not hugely overrepresented and a "T" (the alternative allele) is found at this position. The authors use the correlation between the presence of the T allele and decreased expression of TRPM1 in Fig 5A to support the causal link between this allele and TRPM1 expression. But as this particular polymorphism is closely linked between other polymorphisms in the region it is not clear if this correlation differentiates it from the other nearby polymorphisms? That is, if a similar plot as
shown in Fig5A was made for all of the polymorphisms in the region, would the rs3809579 polymorphism that they focus on stand out as better correlated with transcription than those other polymorphisms?

**Response:** First, all variants in high LD with rs3809579 will indeed show a similar statistical association with TRPM1 expression. This is exactly the main challenge in pinpointing causal variants, and adding the epigenomics layer is critical for this task. At this locus, only rs3809579 overlaps with a LHX2 (and OTX2) peak (and expression of TRPM1 is significantly attenuated upon LHX2 KD). Second, to experimentally evaluate the impact of the C/T alleles on the LHX2 transcriptional activity in this regulatory element, we used a luciferase reporter assay. We examined the luciferase activity of an empty PGL4.3 vector and a vector spanning rs3809579 with either C or T alleles, following transfection with LHX2. Both of these alleles were activated by LHX2 significantly more than the baseline activity of the control empty vector (new Fig. S4, *p* = 0.024, *p* = 0.036 for LHX2 and OTX2, respectively, one-way ANOVA corrected with Dunnett’s for multiple comparisons, N = 6). Importantly, the ratio between the activity of the C and T alleles showed significant elevation, by an average of 25%, for the C compared to the T allele (Fig. 6G, *p* = 0.021, t-test, two-tailed, paired, N = 6). This result shows unequivocally that the C allele increases the transcriptional activity of this region compared to the T allele.

The consensus LHX2 PWM is taken from the Jaspar DB [104] (accession: MA0700.1).

2. The authors then use ATAC-Seq data from heterozygous individuals carrying the two alleles to show that the T allele is less represented than the C allele in the sequencing. This would be expected if this allele were bound less effectively by Lhx2. But it seems possible that it could also be because there are TRPM1 enhancers with variants that are more distally located and therefore the promoter region where the rs3809579 polymorphism might reflect the general transcription differences (with lower ATAC-Seq peaks at promoter regions perhaps correlating with lower transcription). Furthermore, though the authors illustrate in Fig 5D that ChIP-Seq could be used to look at Lhx2 binding between the two variants, the authors have not done this (this should be removed from the schematic). Performing this experiment would provide more direct evidence of a causal link to TRPM1 and would strengthen the conclusion. Secondly, it would be ideal to show using reporter assays that this region with this single polymorphism change has different transcriptional activity.  This would be the prediction based on the manuscript's model and reporter assays allow for the effect of the polymorphism of interest to be directly tested. The ideal cell type would be an RPE cell population.

**Response:** We thank the referee for this suggestion and indeed, as we detailed in our reply to the previous point, in the revised manuscript, we use a reporter assay with the two alleles to establish a causal role for rs3809579.

We have correct the scheme in revised Fig. 6D by removing “ChIP-seq”.

3. In Fig. 4D, this experiment should have negative controls to support the specificity of the interactions. One control is included which is the IP with the Flag antibody on samples only transfected with Otx2 - however as shown in Fig 4D, the Otx2 lane has a lot of background and it is not clear what the result is for this sample. The second control would be to use a control antibody for the IP.

**Response:** We have replaced the image (revised version Fig. 5B) with immunoprecipitation of anti-Flag-LDB1 antibodies following transfection with OTX2, Flag-LDB1, OTX2 and Flag-LDB1, or OTX2 and Flag-LDB1 and LHX2.

OTX2 was detected in the immunoprecipitate only when co-transfected with Flag-LDB1. We did not detect non-specific precipitation of OTX2 by the anti-Flag-LDB1 antibodies when only OTX2, but not LDB1, was transfected into the cells. This shows specificity of the immunoprecipitation experiment and also that the binding of OTX2 and LDB1 are neither dependent on, nor prevented by LHX2.

To clarify this, we have added the following to the Results section (page 12): ” The detection of OTX2 in the Flag-LDB1 immunoprecipitate, with or without LHX2, further supports a direct interaction of human LDB1/2 with OTX2 in hES-RPE. From these results, we infer that LDB1 is directly associated with OTX2 in hES-RPE and that this interaction does not require, and is not inhibited by, LHX2. These findings suggest that LDB1, which is known to interact with LHX2, may mediate the interaction between OTX2 and LHX2, at least on some of the co-bound CREs in the hES-RPE.”

4. Lastly, information about the repeatability of this result is not provided.
   **Response:** The immunoprecipitation of OTX2 by Flag-LDB1 was detected in two independent experiments. Note that this serves to validate the IP-MS analyses which were performed in triplicate on the endogenous LHX2 and OTX2 proteins (Table S9).

Minor concerns
1. Fig 4 - Why is there no interaction detected between Lhx2 and Otx2 through Ldb1? This could use some discussion and also should be tested in their IP experiments (IP with Otx2 in the presence of Ldb1 and Lhx2 and probe for Lhx2 for example - and/or Lhx2 IP and Otx2 probe).

**Response:**

IP-MS was performed on the endogenous proteins in hES-RPE in three independent repeats (Table S9). We validated this by specifically overexpressing the interaction between OTX2 and LDB1-Flag as this is a novel interaction (LDB1 is a known co-factor of LHX2).

Note that co-immunoprecipitation depends on the strength of the antibodies and the extraction and washing buffers. Our protocol eliminates the DNA (incubation with 1% benzonase) because we detected non-specific precipitation without this treatment. The association with the DNA may be required to stabilize the interactions of the transcriptional complex. Further biochemical analyses are underway to decipher the mode of interaction between the proteins that have shared CREs in the RPE. In addition, determining which domain of LDB1 is associated with LHX2 and OTX2 is a topic for future study. We discuss this in the last paragraph of the discussion:

“The interactions between OTX2, LDB1 and SWI/SNF may take place with or without LHX2. Future studies are required to further characterize the proteins’ composition and the physical interactions between them (OTX2 and LHX2 or OTX2 alone, with or without LDB1 and SWI/SNF), and to further determine how this impacts the transcriptional output and its relevance to retinal disease mechanisms”

2. Pg3 "…as for uncovering mechanism…" needs to be rephrased

**Response:** This section has been revised:

“Mapping tissue-specific cis-regulatory elements (CREs) of the RPE and the TFs responsible for these regulatory elements’ selection is fundamental for understanding the mechanisms governing acquisition and maintenance of cell-specific transcriptional programs and for resolving the contribution of the CREs to complex diseases, such as AMD”

3. Pg 4 "Among these the Orthodenticle…" Incomplete sentence?

**Response:** This has been corrected (page 4):

**“**Among these, Otx2 the paired-type homeodomain (HD) TF, is essential for patterning of anterior neural plate and subsequent formation of forebrain, midbrain and early eye primordia, and is also required later in development for RPE differentiation [8-12].”

4. Pg 4 "Otx2 is also maintained in the RPE in…where it required…" needs to be rephrased

**Response:** This was changed from “Otx2 is also maintained in the adult RPE in the adult, where it required for the expression of numerous RPE genes, a function attributed to its regulation of and interaction with the microphthalmia-associated transcription factor (Mitf). The latter is a basic domain helix–loop–helix leucine zipper (bHLH-LZ) TF that is required for the specification and differentiation of melanocytes and ocular pigmented cells (ref..)”

to:

“Otx2 is also maintained in the adult RPE, where it is required for the expression of multiple RPE genes [13-16]. Activity of Otx2 in RPE is attributed to its regulation of, and interaction with the microphthalmia-associated TF (Mitf), a basic domain helix–loop–helix leucine zipper (bHLH-LZ) TF that is required for the specification and differentiation of melanocytes and ocular pigmented cells [15-19].”.

5. Pg 4 - "…Lim HD TF Lhx2, the orthologue…" Ortholog or paralog?

**Response:** Changed to “homolog”

6. Pg 5 - a preview of the results to be shown in the paper could be useful here.

**Response:** We now include the following preview:

“Here we studied Lhx2/LHX2 in developing mouse and human RPE generated from stem cells (hES-RPE). Through functional and genomic analyses, we reveal that LHX2 functions upstream and together with OTX2 on shared genomic regions (cistrome) in regulation of target genes. Through this feed-forward regulatory module LHX2-OTX2 control the RPE transcriptional program. Proteomic analyses further exposed co-factors of LHX2 and OTX2 that likely mediate the tissue-specific gene regulation, including LDB1 and the SWI/SNF chromatin remodeling complex. Finally, intersecting the map of the LHX2-OTX2 bound cistrome with published genomic data on AMD revealed a causal non-coding risk-SNP that acts by altering TRPM1 expression in the RPE through the modulation of LHX2 binding to its promoter. The study exemplifies how delineation of tissue-specific transcriptional regulators, their cistromes and downstream gene-regulatory networks can provide insights into a complex disease’s pathology.”

7. Dapi signal of E16.5 (J) - not very clear image

**Response:** The image in Fig. 1J conveys the reduced expression of E-cad and OTX2 in the RPE with conditional deletion of Lhx2. The reduced expression of these markers precludes detection of the cell membrane and this likely contributes to the “fuzzy” appearance. We believe that the image conveys the reduced expression and hope that it can be retained.

8. Pg9 "…two enhancers associated with the OTX2 gene…" Is there evidence to suggest that they are indeed enhancers? That is have they been tested for reporter activity in one of the many reports of cis-regulatory elements in the Otx2 region?

**Response:** We have changed “enhancers” to “CREs”. One of these regions, hs1150 {Visel, 2007 #6465}, was previously documented to be active in mouse RPE {Bhansali, 2020 #12968}. We conducted a luciferase reporter assay on this region and the results support its activation by OTX2 and LHX2; this activity is further enhanced by the overexpression of both (Fig. 4D) and see detailed response to reviewer #2 comment 2).

9. Pg 10 "… by which LHX2 activate gene expression…" needs to be rephrased
**Response:** This sentence has been replaced with “is important for transcriptional activity of LHX2 in RPE”

10. Pg 11 "… - a known co-factor of LDB1 complexes…" no reference for this statement
**Response:** References have been added.

{Chen, 2002 #502;Wang, 2020 #503}

11. Pg 12 "… and that their activity, specifically that of Otx2 - …" Not sure what this phrase (and the whole sentence together) means  - it needs to be clarified and referenced if there is a reference.

**Response:** The above sentence was replaced by:

“Overall, the results from the IP-MS proteomic analyses suggest that the interaction between LHX2 and OTX2 is mediated through LDB1/2 (Fig. 5C). The detection of direct interactions of OTX2 with the BAF complexes suggests a chromatin-remodeling activity for OTX2 and its bound co-factors, allowing robust transcriptional activation of multiple tissue-specific genes (scheme of proposed transcriptional module and co-factors, Fig. 5C). “

12. Pg 12 "…from numerous distal RPE enhancers that control the RPE specific transcriptional output" If this refers to the data that is only from this paper this seems to not be well supported. There is no evidence that these elements drive specific transcription or that they aren't also active in other cell types or that the BAF complex is critical for some or any activity.

**Response:** We replaced this with the following: “The numerous genomic regions bound by both LHX2 and OTX2 and reduced expression of the genes associated with these regions (based on proximity) suggest that these factors function together on CREs to regulate gene expression in the RPE.””

Also, as explained in response to reviewer #2 comment 2, we now add results that support LHX2 and OTX2 co-activation of an enhancer associated with Otx2 regulation (new Fig. 4C, D).

We are currently in the process of analyzing the BAF conditional mutants in the RPE. The findings support a key role for SWI/SNF in RPE differentiation (manuscript in preparation).

13. Pg 13 "This SNP is very common…" It seems most likely this refers to the T allele, but this should be stated more clearly.

**Response:** Typically, the “commonness” of a SNP refers to the frequency of its minor (less common) allele. As we state in the relevant section (page 14 in the revised manuscript), in the European population, the minor allele of this SNP is the C allele, and its frequency is 42% (the T allele is the more prevalent one).

14. Pg 15 "…that if acts by altering TRPM1…" "it" instead of "if"

**Response:** Corrected

15. Pg 15 "Thus, the regulation of the Trpm1 locos…" should be "locus"

**Response:** Corrected

16. Pg. 17 - "…reveals an important role for LDB1 in mediating the interaction between LHX2 and OTX2". This statement is not formally supported as there is no data to support that they are in fact in a complex. Could it be that they bind in a mutually exclusive manner (to the same domain of LDB1 for example)?

**Response:** We have deleted this text. We agree that further analysis is required (and it is underway) to address the domains that mediate the interactions and their impact on the transcriptional activity. This is now addressed in the last paragraph of the discussion.

17. Pg 18 "There could be several complexes, and future studies…" At some point, in the discussion perhaps, it would be good to discuss the possibility of Crx as occupying some of those "Otx2 sites" Crx has been identified to be expressed in the RPE - this is one of the best characterizations : <https://academic.oup.com/hmg/article/18/1/128/614283>

**Response:** We have added this reference to the revised introduction, where we address Otx2’s importance in the adult RPE (page 4). Because we did not examine Crx in this study, further discussion of its possible involvement is beyond the scope of the current work.

18. Fig 5A - this data needs to be described better, even though it seems to be reproduced from a previous publication - one cannot interpret it as it is currently presented.

**Response:** We have tried to explain these data better in the revised text:

“In accordance with higher LHX2 binding affinity and thus stronger promoter activity for the C allele, eQTL analysis, based on the resource provided by [48] indeed shows that this allele is associated with higher expression level of TRPM1 compared to the T allele (Fig. 6A).”

19. Pg 20 "Two vectors, which showed the strongest effect…" The metric that is being used and the assay to measure should be described.

**Response:** We have added the following explanation to the Methods:

“Downregulation of LHX2 in hES-RPE was performed using pGIPZ vectors (Thermo Fisher Scientific) for targeting LHX2 mRNA (NM_004789). The efficiency of four vectors was examined in D407 cells following transduction and 48 h of incubation. We examined the knockdown efficiency by quantitative PCR. The two vectors that showed the strongest effect, of 75% (V2LHS_35479, mature antisense AATGGCAAAGTAAGACTTC; and V3LHS_319912, mature antisense AGATGCTACCGTCCTTGCT) were used for LHX2 knockdown (KD) in hESRPE For the negative control, we used non-silencing (NS)- shRNA pGIPZ vector (RHS4531).”
