# Supplementary material for: The LHX2-OTX2 transcriptional regulatory module controls retinal pigmented epithelium differentiation and underlies genetic risk for age-related macular degeneration
Source: PLoS Biol. 2023 Jan 17;21(1):e3001924. doi: 10.1371/journal.pbio.3001924 (PMC9844853; doi:10.1371/journal.pbio.3001924)
Supplement: Attachment — Submitted filename: rebuetell letter_3rd revision_9112022.docx [file pbio.3001924.s017.docx]

We thank reviewer 4 for revaluation of the our revised manuscript. The reviewer raised few questions concerning the reporter assay. We thank the reviewer for the comments, which allowed us to clarify and improve the presentation and explanation of the analysis and results of the reporter assay. We have fully addressed the concern concerning the assay and this is explained point-by-point below.

1) *The main text suggests that the results shown in Figure 6G are under conditions where Lhx2 is overexpressed. But the figure legend or the figure mark-up makes no mention of this. This should be clarified.*

**Response:**  To clarify this we added to the figure legends

Fig 6 “Luciferase assay for comparing the effect of the two alleles of rs3809579 on the activity of the regulatory element bound by LHX2 in the promoter of TRPM1 **following LHX2 overexpression**.”

Fig. S4 “Luciferase reporter activity induced by LHX2 **overexpression** on 309 bp that contain regulatory element bound by LHX2 in rs3809579.”

In the methods section it is mentioned that the luciferase assay is conducted by over expression. Note that each data point represents average of three technical repeats. We now also added the following information:

“**Each data point represent average of at least two technical replicates. The number of biological repeats is indicated in figure legends**.”

*2) Assuming that Figure 6G and SuppF4 both use Lhx2 misexpression, why are these data plotted differently - Supp4F shows a bar graph with the (empty) basal vector data in its own column. But Figure 6G does not have this data shown and instead has the empty vector factored into the data, as suggested by the y-axis notation. How was this done? Were samples randomly paired together? Was a separate set of controls done for each? Are the datapoints shown in Figure 6G the same as those shown in SuppF4? If a statistical test was done to directly compare the T and C variant activity shown in SuppF4, would these be statistically different? If not, does this suggest that the evidence for a functional difference in Fig 6G is not solid?*

**Response:** We thank the reviewer for this comment, which allowed us to clarify and improve the presentation and explanation of the analysis and results of the reporter assay.

Yes, Figure 6G and Fig. S4F show the results of the same experiment (and are based on the same data), but have different aims: Figure 6G demonstrates that the TRPM1 construct with the C allele has higher transcriptional activity than the one with the T allele. Fig. S4F aims to show that both constructs (with either C or the T alleles) have increased activity compared to the empty PGL4 (basal) vector. That is, it serves to show that this element indeed functions as an enhancer.

In each of the reporter experiments, we compared luciferase activity, following over-expression of LHX2, from the following three vectors; empty PGL4.23, PGL4.23 with CRE-TRPM1-C, and PGL4.23 with CRE-TRPM1-T. The Renilla vector was included in all transfections to normalized for the transfection efficiency. Importantly, this experiment includes six independent replicates (done on different days or by different students), where each replicate contained two or three technical repeats. (Each dot in the plot is an average of the technical repeats).

The statistical analysis was done on paired samples from the same experiment (which were done on different days or by different students). This is essential to account for variability which is not due to LHX2 activity (like passage of the cells, lot of transfection reagents, calibration of the luciferase reader etc.).

Yes, the difference between activity of the T and C allele shown in Fig. S4F is statistically significant too (when a paired sample is used, as it should; note, it is based on the same data shown in Figure 6G and therefore the difference is significant, like 6G). Given the referee’s comment, we realized that we used a graph that does not represent the paired-sample design of the experiment and therefore obscure the difference between the T and C allele. We now use a graph that fits better the experimental design, and makes the difference between T and C much more evident. In all 6 experiments, transcriptional activity (a) of the CRE-TRPM1-T construct is higher than empty and (b) the CRE-TRPM1-C is higher than CRE-TRPM1-T (overall p-value of this consistent trend is 0.5^6^=0.016; binomial test). We revised the legend of Fig. S4

“Luciferase reporter activity induced by LHX2 overexpression on 309 bp that contain regulatory element bound by LHX2 in rs3809579. Both CRE-TRPM1-T and CRE-TRPM1-C alleles are significantly activated by LHX2 as compared to its activation of empty. The p-values calculated by One-way ANOVA, matched samples, corrected with Dunnett’s for multiple comparisons (N=6). In all 6 independent experiments, transcriptional activity of the CRE-TRPM1-C is higher than CRE-TRPM1-T (overall p-value of this consistent trend is 0.5^6^=0.016; binomial test).”

We add the excel file (Table S10) with the raw data, normalization and statistical analyses.

The table contains two tabs:

CRE_OTX2_Fig4_S3: The Luciferase activity of the CRE-OTX2 following over expression of LHX2 or OTX2 or both. The raw data, normalizations and statistical analyses are included.

TRPM1C-T_ Fig6_S4: The Luciferase activity of the CRE-TRPM1-T or CRE-TRPM1-C following over expression of LHX2. The raw data, normalizations and statistical analyses are included.

*3) The variability of the datapoints in SuppF4 suggests that reproducibility is an important issue. How many times has this experiment been repeated? This should be described.*

**Response:**  As we noted above, our previous plot reflected technical variability that is not related and not relevant to the factor-of-interest of this experiment (the T vs. C allele). When testing paired samples, in fact, the effect (higher activity of the C allele) is very reproducible and appears in all 6 replicates.

*Can the authors provide a rationale for why they need to add Lhx2 to the mix to get this result - is there Lhx2 in these cells already, and if so, why isn't that sufficient to activate the reporter?*

**Response:** Note that the effect of GWAS common risk SNP on gene expression is expected to be relatively small (in contrast to mutations underlying Mendelian traits). We used overexpression to assure activity of LHX2 on two allele will be high enough for detecting the difference between them. Reassuringly, we did observe consistent and significance difference in the activation of the two alleles by LHX2 (see revised Fig S4, Fig. 6). This strongly support the impact of the C/T variant on the transcriptional activity.

*Can reporters for the C and T alleles be introduced into the same cells so that sample variability is decreased and is instead controlled internally? Does the luciferase system allow one to use renilla to be driven by one of the variants and luciferase by the other (instead of renilla being driven by an unrelated element).*

**Response:**

Dual luciferase reporter assay is employed by numerous laboratories the vectors we used are optimized expression yet limited background activity in mammalian cells.

We agree that other reporter systems are required, especially when we aim at high throughput analyses. We are considering alternatives, possibly based on sequencing technologies but this is beyond the scope of the current study and will be implemented in future studies.
